# Ectopic Expression of a *Neospora caninum* Kazal Type Inhibitor Triggers Developmental Defects in *Toxoplasma* and *Plasmodium*

**DOI:** 10.1371/journal.pone.0121379

**Published:** 2015-03-24

**Authors:** Zoi Tampaki, Ramadhan S. Mwakubambanya, Evi Goulielmaki, Sofia Kaforou, Kami Kim, Andrew P. Waters, Vern B. Carruthers, Inga Siden-Kiamos, Thanasis G. Loukeris, Konstantinos Koussis

**Affiliations:** 1 Institute of Molecular Biology and Biotechnology, Foundation for Research and Technology—Hellas, Heraklion, Greece; 2 Department of Biology, University of Crete, Heraklion, Greece; 3 Department of Medicine, Albert Einstein College of Medicine, Bronx, United States of America; 4 Faculty of Biomedical & Life Sciences, Glasgow Biomedical Research Centre, University of Glasgow, Glasgow, Scotland, United Kingdom; 5 Department of Microbiology and Immunology, University of Michigan Medical School, Ann Arbor, United States of America; Institut National de la Santé et de la Recherche Médicale—Institut Cochin, FRANCE

## Abstract

Regulated proteolysis is known to control a variety of vital processes in apicomplexan parasites including invasion and egress of host cells. Serine proteases have been proposed as targets for drug development based upon inhibitor studies that show parasite attenuation and transmission blockage. Genetic studies suggest that serine proteases, such as subtilisin and rhomboid proteases, are essential but functional studies have proved challenging as active proteases are difficult to express. Proteinaceous Protease Inhibitors (PPIs) provide an alternative way to address the role of serine proteases in apicomplexan biology. To validate such an approach, a *Neospora caninum* Kazal inhibitor (NcPI-S) was expressed ectopically in two apicomplexan species, *Toxoplasma gondii* tachyzoites and *Plasmodium berghei* ookinetes, with the aim to disrupt proteolytic processes taking place within the secretory pathway. NcPI-S negatively affected proliferation of *Toxoplasma* tachyzoites, while it had no effect on invasion and egress. Expression of the inhibitor in *P*. *berghei* zygotes blocked their development into mature and invasive ookinetes. Moreover, ultra-structural studies indicated that expression of NcPI-S interfered with normal formation of micronemes, which was also confirmed by the lack of expression of the micronemal protein SOAP in these parasites. Our results suggest that NcPI-S could be a useful tool to investigate the function of proteases in processes fundamental for parasite survival, contributing to the effort to identify targets for parasite attenuation and transmission blockage.

## Introduction

The phylum Apicomplexa comprises a number of intracellular parasites causing disease in humans and animals. Two prominent members are *Plasmodium* parasites causing malaria and *Toxoplasma* that is the causative agent of toxoplasmosis in immunocompromised individuals. These parasites are characterized by having both invasive and replicative forms. In *T*. *gondii* during the asexual life cycle tachyzoites invade cells and replicate inside a parasitophorous vacuole in the host cytoplasm. Newly formed parasites egress from the host cell, and immediately invade new target cells. During these events the secretory organelles of tachyzoites, micronemes, rhoptries and dense granules (DG) have been found to have important roles. *Plasmodium* parasites also have a complex life cycle. The parasite goes through a replicative cycle in the blood of the human host, causing the pathology of the disease. *Plasmodium* is transmitted by mosquitoes. After the uptake of sexual forms in a blood meal, the parasite develops into a zygote in the mosquito midgut. The zygote in turn matures into the motile ookinete, which traverses the midgut epithelium and forms the sporogonic oocyst. Sporozoites having developed inside the cyst are transmitted to a new host during a blood meal. Zygote to ookinete transition is a crucial point in the life cycle of the parasite as a failure to successfully complete this step blocks transmission. This transition is accompanied by a radical reorganization of the cell, including formation of the micronemes, which have an important role in motility and invasion, and an extension of the cell through the elongation of the cytoskeleton.

Proteases have been recognized as basic components in the life cycle of apicomplexan parasites regulating a plethora of physiological processes such as replication, host invasion, egress and metabolism [1–4]. Apart from increasing our understanding of the basic biology of apicomplexans, proteases comprise potential targets for drug development [5] or for interventions aiming at parasite attenuation or transmission blockage.

Serine proteases have been identified in both *Plasmodium* and *Toxoplasma* and primarily subtilisins and rhomboids have been studied in more detail. In *T*. *gondii* 12 genes encoding subtilisin-like proteases have been identified, [6–9], while in *Plasmodium*, three subtilisin-like proteases have been found [10–13]. In the case of rhomboids, 6 are encoded in *T*. *gondii* and 8 in *Plasmodium spp* [14,15]. Genetic studies have shown that many of these proteases are essential [6,8,16–20]. However, with the exception of *Plasmodium* SUB1, *in vitro* biochemical assays to further elucidate their function, identify potential substrates and develop potential inhibitors are either not available or technically challenging with contradictory data in some cases between *in vitro* and *in vivo* studies [21,22].

Small molecule protease inhibitors (SM-PIs) with a broad range of activity have been employed in different experimental models to uncover the significance of regulated proteolysis in developmental and physiological processes. SM-PIs have been also tested against apicomplexan parasites revealing the overall importance of proteolysis in host cell invasion, egress and intracellular parasite replication [23–26]. Among the disadvantages of SM-PIs are that they affect in parallel proteolytic processes taking place in different cellular compartments (including proteolysis in the host cells) and/or involving different types of proteases.

On the other hand Proteinaceous Protease Inhibitors (PPIs) are considered superior inhibitors to SM-PIs. These molecules have evolved as one of the various self-protecting strategies against assaulting or uncontrolled proteolysis. Firstly, PPIs are more specific inhibitors than SM-PIs since co-evolution with their target proteases has shaped their specificity, and secondly because PPIs are proteins their expression/activity can be restricted in a specific stage and/or in a specific subcellular compartment.

Serine PPIs can be distinguished based on their structure and their mechanism of action to serpins, canonical or non-canonical inhibitors [27]. A well-studied family of PPIs inhibiting serine proteases is that of Kazal type inhibitors. The basic domain of Kazal inhibitors has a characteristic structure dictated by six conserved cysteines forming intra-domain disulfide bonds [28]. Non-canonical inhibitors such as hirudin are much less abundant; they occur only in blood sucking organisms and inhibit proteases involved in clot formation [29,30].

The distribution of PPIs in parasites varies. While *Neospora* and *Toxoplasma* have canonical Kazal type inhibitors as well as serpins, other apicomplexan parasites such as *Plasmodium* and *Babesia* do not possess any serine protease inhibitors. A protective role against digestive enzymes has been hypothesized for the four-Kazal domain inhibitors, TgPI-1 and TgPI-2, identified in *Toxoplasma* [31,32]. Besides putative homologues of TgPI-1 and TgPI-2, *N*. *caninum* expresses the single Kazal domain NcPI-S that strongly inhibits bacterial subtilisins *in vitro* [33,34] and it has been speculated that NcPI-S might regulate endogenous subtilisin activity in *N*. *caninum* [34]. Apart from their difference in the number of Kazal-domains, there is no similarity in the amino acid composition of the active sites of these inhibitors [34] suggesting that they possess distinct substrate specificities.

We investigated the potential of NcPI-S to interfere with proteolytic processes within the secretory apparatus, by ectopically expressing NcPI-S in *Toxoplasma* and *Plasmodium*; neither of which express an NcPI-S homologue. *Toxoplasma* tachyzoites provided our study with a cellular and biochemical environment equivalent to that of the native NcPI-S, as the two species *T*. *gondii* and *N*. *caninum* are phylogenetically very close. This strategy provided a system for a fast evaluation of the chosen inhibitor. After confirming that active NcPI-S affected the growth of *T*. *gondii* tachyzoites, we designed a stage specific ectopic expression of NcPI-S in the rodent model parasite *P*. *berghei* that resulted in an arrest of zygote to ookinete transition. Our results indicate that PPIs can be used as tools to study parasitic proteases and additionally could lead to the discovery of novel antiparasitic agents.

## Materials and Methods

### Ethics Statement

All animal work has passed an ethical review process and was approved by the FORTH Ethics Committee (FEC). All work was carried out in full conformity with Greek regulations: Presidential Decree (160/91) and law (2015/92) which implement the directive 86/609/EEC from the European Union and the European Convention for the protection of vertebrate animals used for experimental and other scientific purposes and the new legislation Presidential Decree 56/2013. The experiments were carried out in a certified animal facility license number EL91-BIOexp-02.

### Parasites, cells, mosquitoes

Human foreskin fibroblasts (HFFs—ATCC SCRC-1041) were maintained in Dulbecco's Modified Eagles Medium (DMEM) containing 10% heat inactivated Fetal Bovine Serum (FBS, PAA Laboratories), 2 mM Glutamine, 1% Penicillin/Streptomycin (GIBCO) (D10 Complete). The *T*. *gondii* RH strain and the RHΔhxgprt [35] were maintained by serial passage in HFFs as described [36]. Tachyzoites were harvested after lysis of the monolayer, unless otherwise stated, by scraping, passage through a 26-gauge needle and a 3-μm Nucleopore membrane filter (Whatman), and collected by centrifugation (400xg, 15 min at 4°C). *P*. *berghei* strains were ANKA HP (also called 15cy1A), and the GFP-expressing 507cl1 clone [37]. *Anopheles gambiae* G3 strain was used for mosquito infections.

### Plasmid constructions

All primers are listed in S1 Table in supplemental material.

#### pGra1/ssmycNcPIS

ROP1 signal sequence (ss) was amplified from pRop1myc plasmid [38], using primers ssRop1_5’and ssRop1_3’.The myc tag was amplified using mycRI5’ and myctoxo3’ primers. NcPI-S was amplified from pQE/NcPIS [34], using NcPIS_5’ and NcPIS_3’ primers. The fragments were fused in frame and inserted in pGra1/GFP/Gra2-SK [39], which contains the dhfr_5/HXGPRT/dhfr_3 selection marker, using the NsiI and PacI restriction sites (S1A Fig.).

#### pGra1/ssmycNcPISmut

NcPISmut was generated from NcPI-S by PCR-based mutagenesis with primers NcPIsFor and NcPISRev. The construct encoded NcPI-S with changed amino acids P2, P1 and P1’-P4’ of the Kazal inhibitory domain from SMEYDP to FASGKR. The construct was verified by sequencing. For cloning in the transfection vector NcPIsmut was amplified with the primers Ncmut_5’ and Ncmut_3’ and subcloned into FseI-PacI digested pGra1/ssmycNcPIS resulting in the exchange of the wild type NcPI-S with the mutant version.

#### p[CTRP-Sp-V5-NcPIS]DEFSSUToxo

The signal sequence of the PbSUB2 was amplified from *P*. *berghei* gDNA with primers SPsub-2F and SPsub-2R. The V5 epitope was amplified from the pIZ/V5-His vector (Invitrogen) using V5for and V5Rev primers. NcPI-S (excluding the signal sequence) was amplified from the pQE/NcPIS plasmid with primers NcPIS_5’ and NcPIS-R. The fragments were joined in an intermediate vector and subsequently inserted between the CTRP promoter and P28 3’ UTR in the vector pCp∼S [40]. The derived [CTRP-Sp-V5-NcPIS] cassette was finally inserted into the pDEF-TgDHFR/D-SSU (RV) transformation vector (kindly provided by Dr. Franke Fayard).

#### NcPISmut

NcPI-Smut was inserted in the p[CTRP-Sp-V5-NcPIS]DEFSSUToxo using FseI and PacI/NotI restriction enzymes. The sequences encoding the PbSUB2 signal sequence and V5 remained the same.

### Transfection, selection and cloning of stable transformants

To generate stable transformants freshly released RHΔhxgprt parasites were electroporated as described [41] with 100 μg of Gra1/ssmycNcPIS and Gra1/ssmycNcPISmut constructs. Tachyzoites were transfected in presence of NotI restriction enzyme [42] and parasites were selected with mycophenolic acid and xanthine as described [43]. Resistant parasites were cloned by limiting dilution. Clones NcPI-S_6 and NcPI-S_8 from two independent transfection experiments, and clone NcPI-Smut_16 were selected.

*P*. *berghei* schizonts were transfected using standard procedures [44]. Pulse Field Inversion Gel Electrophoresis (PFIGE) and genomic PCR genotyping was used to characterize the transgenic clones (S2B, S2C Fig.). For PFIGE-Southern, total gDNA was separated on 1% agarose gel under Pulsed-Field Inversion Gel Electrophoresis (PFIGE) conditions, transferred and hybridized with *P*. *berghei* radio-labeled *dhfr-ts* 3’UTR probe as described [44] (S2B Fig.). Genomic PCR genotyping was performed using integration specific primer sets [45]: L665 and L740. Clone purity and determination of the integration site (*c* or *dssu* locus), were verified by using forward primers *cssu* specific L270 and *dssu* specific L260 in combination with the reverse L740 primer common to both loci [46] (S2C Fig.).

Two independent clones expressing NcPI-S were studied; *NcPIS_3*, in the *GFP507cl1* (*GFPp*) recipient strain [37] and *NcPIS_1*, the latter derived from a transfection using the ANKA 15cy1A reference strain as a recipient. The *NcPISmut* line expressed the mutated form of NcPI-S and was derived from the *GFP507cl1* (GFPp) recipient strain. The strain ANKA 2.34 was used as a WT control in oocyst experiments.

### *T*. *gondii* assays

Equal numbers of freshly released filtered tachyzoites were used to infect HFF cells during replication assays. After sedimentation and incubation for 1 h, cultures were washed and incubated for another 24 h. Coverslips were labeled with anti-SAG1 antibody and 4'-6-Diamidino-2-phenylindole (DAPI). Vacuoles containing different numbers of parasites (1, 2, 4, 8 or 16) were counted (total 150 vacuoles/sample). Three independent experiments in duplicates were performed.

Invasion competence of the parasites, was determined according to previously described protocols [47]. Freshly lysed parasites from all strains were allowed to invade HFF cell monolayers grown on coverslips for 10 min and the number of extracellular vs. intracellular parasites was determined. Extracellular tachyzoites were revealed after labeling with anti-SAG1 without permeabilization and intracellular tachyzoites with subsequent staining with anti-GRA3 after permeabilization.

The percentage of egressed vacuoles was determined after inducing egress with calcium ionophore A23187. Intracellular replicating tachyzoites (36–40 h p.i.) were treated with 1 μM A23187 or DMSO (solvent control) for 5 min [48] and egressed vacuoles were enumerated in each sample.

### *P*. *berghei* assays

*P*. *berghei* ookinete cultures and purification was performed as previously described [49]. Conversion rates were calculated as previously described, using a mouse monoclonal antibody (13.1), which recognizes the P28 protein on the surface of female gametes, zygotes and ookinetes [50]. Positive cells were counted on a Zeiss Axioscope2plus microscope (Carl Zeiss, United States) equipped with an AxioCam color Zeiss CCD camera. For oocyst counting, mosquitoes were fed on anaesthesized infected mice for gametocyte feeding. Alternatively, membrane feeders were used for feeding *in vitro* cultured ookinetes. Oocysts were counted on day 10 post-feeding.

### Transmission Electron Microscopy

For TEM observations, ookinetes were pelleted at 720xg and fixed in 2% glutataraldehyde/2% paraformaldehyde in 0.1 M sodium cacodylate buffer pH 7.4 for 45 min at 4°C, post fixed in 1% osmium tetroxide, dehydrated in graded ethanol, stained with uranyl acetate 1% and finally embedded in graded Durcupan:propylene oxide series (1:3, 1:1, 3:1, 4:0). Ultrathin-sections (50–100nm) were taken on a Leica LKB2088 ultramicrotome and examined under JEM 100C/JEOL/Japan Transmission Electron Microscope. Microphotographs were obtained with an ES500W Erlangshen camera and analysed by the DigitalMicrograph software (Gatan, Germany).

### Antibodies

For immunoblotting of *T*. *gondii* the antibodies were: 9B10 anti-myc (1:5000 dilution) (Cell Signaling Technology), anti-MIC2 (6D10) [51], anti-SAG1 (gift of Jean Francois Dubremetz), anti-ROM4 (gift of Dominique Soldati-Favre) and anti-GRA1 (gift of Louis Weiss), all 1:1000 dilution. For Western blot experiments of *P*. *berghei* anti-V5 (Invitrogen, diluted 1:2000), rabbit anti-SOAP (developed in the Loukeris laboratory, diluted 1:500), the monoclonal antibody 13.1, which detects the P28 surface antigen of *P*. *berghei* ookinetes [52] (1:20000), anti-Bip (1:2000-gift of Ellen Knuepfer, NIMR, UK) were used. For IFA of *T*. *gondii* mouse a-myc 9B10 (1:2000, Cell Signaling Technology), rabbit anti-GRA3 (1:300, gift of Jean Francois Dubremetz), rabbit anti-PfBip (MR4 ATTC, MRA-20 1:1000) were used. Antibodies used for IFA of *P*. *berghei* were anti-serpin6 (1:1000, provided by G. Christophides) and PbCAP380 that recognizes the oocyst capsule [53]. Secondary antibodies were conjugated to Alexa 555 or Alexa 488 (Molecular Probes).

### Fractionation and Immuno blot analysis

For analysis of *T*.*gondii* one infected HFF culture was left to lyse spontaneously, and extracellular tachyzoites were purified from the culture medium. For a second parallel culture, intracellular tachyzoites were force released (20–22 h p.i.) by trypsinolysis, passage through a 26-gauge needle, and filtering through a 3 μm Nucleopore membrane filter. Tachyzoite pellets were resuspended in Tris-Cl pH 8.5, lysed by freeze-thaw and sonication, centrifuged for 30 min at 320xg, and the supernatant was removed (Soluble fraction, S). The pellet was resuspended in 0.1 M Na_2_CO_3_ pH 11.5, incubated on ice for 1 h, and centrifuged at 320xg for 30 min in order to remove peripheral membrane proteins. The supernatant (Salt-eluted fraction, SE) was separated from the remaining pellet that was resuspended in PBS (Membrane fraction, M). β-mercaptoethanol was added to the lysates and samples were separated on 15% SDS-PAGE gels and transferred to nitrocellulose membranes.

For detection of NcPI-S in *P*. *berghei* zygotes/ookinetes, parasites were resuspended in 10 volumes of RIPA buffer (50 mM Tris-Cl pH 8.0, 150 mM NaCl, 2 mM EDTA, 1% NP-40, 1% Sodium Deoxycholate, 0.1% SDS) supplemented with Protease Inhibitor Cocktail (Sigma). After incubation on ice for 30 min and centrifugation 8000xg for 30 min, the cleared lysates were separated on SDS-PAGE gels and processed for Western blot analysis. For *P*. *berghei* fractionation, parasite pellets were initially resuspended in 10 volumes of 100 mM Tris-Cl pH 8.0 and incubated on ice for 1 h. After centrifugation at 8000xg for 30 min, supernatants were collected, while the pellet was resuspended in 10 volumes of RIPA buffer, loaded on SDS-PAGE gels and processed for Western blot analysis. To detect phosphorylation of PbeIF2a in the different strains, parasite pellets were resuspended in 10 volumes modified RIPA buffer (50 mM Tris-Cl pH 8.0, 150 mM NaCl, 2 mM EDTA, 1% NP-40, 0.1% SDS) supplemented with Protease Inhibitor Cocktail (Sigma), 50 mM NaF and 1mM phenylmethylsulfonyl fluoride (PMSF). Subsequent treatment was as described above.

### Indirect immunofluorescence microscopy

All manipulations were carried out at room temperature. *T*. *gondii* tachyzoite-infected HFF cells on 24-well chamber slides were fixed with 100% cold methanol for 10 min, followed by three washes in 1xPBS. Fixed cells were blocked in 1% bovine serum albumin (BSA) (Merck, Europe) with 0.25% TritonX-100 in PBS for 1 hour. The wells were then stained with different primary antibodies, followed by conjugated secondary antibodies. DAPI was added to stain the nucleus. Images were collected using a Zeiss Axioscope2plus (Carl Zeiss, United States) microscope equipped with an AxioCam color Zeiss CCD camera. Images were processed using ImageJ software. For *P*. *berghei* ookinete IFAs, purified ookinetes were allowed to settle on glass slides pre-treated with poly-L-lysine for 15–30 min. The following procedures were all performed at RT. Samples were fixed in 4% paraformaldehyde (PFA) for 10 min and washed with PBS. This was followed by permeabilisation in PBS, 0.1% triton-x100 (PBT) for 30 min. The primary antibody directed against V5 was added, after 1 h followed by PBS washes and incubation with secondary antibody for 30–45 min. Nuclei were stained with TO-PRO. Samples were washed in PBS and mounted in Vectashield before being analyzed using a Leica TCS SP2 confocal laser scanning microscope. For mosquito midgut IFA, infected midguts were dissected in ice-cold PBS, prefixed for 90 sec in ice-cold 4% PFA in PBS, transferred to ice-cold PBS and cut open longitudinally. After removal of the midgut content the epithelium was fixed for another 45–60 min in 4% PFA at RT. Samples were washed 3x10 min in PBS, blocked and permeabilized for 1 h 30 min in 1% BSA in PBT at RT, followed by incubation overnight at 4°C with anti-serpin6, followed by 3 washes with PBT and incubation with secondary antibodies for 1 h at RT. Nuclei were stained with TO-PRO 3 (Molecular Probes). Midguts were washed three times in PBT for 10 min, mounted in Vectashield and analyzed as described above.

### *In vitro* inhibitory activity of transgenic parasite lysates

Increasing amounts of lysates (calculated as parasite number equivalents) prepared from RH and NcPIS_6 were incubated with *Bacillus licheniformis* subtilisin A (*Bl*-subA) (3.6 nM) in reaction buffer (100 mM Tris–HCl, 150 mM NaCl, 10 mM CaCl_2_, Triton X-100 0.25% v/v, 0.05% sodium Deoxycholate, pH 8.0) for 15 min at 37°C followed by the addition of the substrate CBZ-Gly-Gly-Leu-pNA (0.15 mM). Residual hydrolytic activity (R.H.A) was measured as substrate conversion in a spectrophotometer. No enzymatic activity was detected when lysates were incubated with the substrate alone (NcPIs_6/NP) or with *Bl*-subA in presence of 2mM PMSF (NcPIS_6/+PMSF).

### Statistical analysis

Student’s t test using SPSS v.11.5 software was employed.

## Results

### NcPI-S expression in bacteria, NcPI-S modifications and their validation

**Fig 1:**
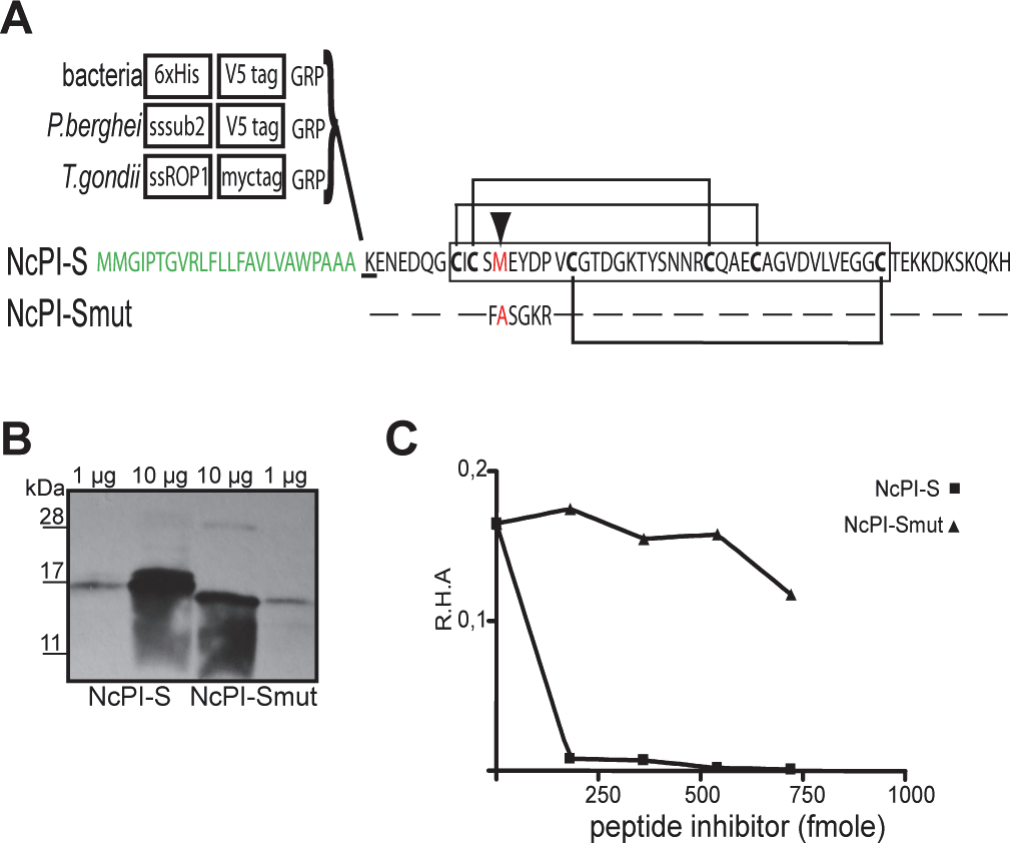
Bacterial expression of NcPI-S. (A) Amino acid sequence of NcPI-S. The deduced signal peptide sequence is shaded in green and the signal peptidase cleavage site is underlined. An open box indicates amino acid sequence of Kazal inhibitory domain. The methionine (M) in position P1 is shaded in red and the conserved cysteine residues are in bold. Also depicted are the putative intra-domain disulfide bridges between cysteine numbers 1–5, 2–4, 3–6. Native signal peptide of NcPI-S (without the peptidase cleavage site) was replaced by a 6xHis and a V5 tag (in the case of bacterial expression), by the signal peptide of ROP-1 and a myc-tag (*T*.*gondii* expression) and by the signal peptide of PbSUB2 and a V5-tag (*P*.*berghei* expression). Ectopic expression of NcPI-Smut in all cases was accomplished by exchanging NcPI-S sequence with that of NcPI-Smut, where residues P2-P4’ have been mutated. (B) Western blot analysis of different amounts (1–10 μg) of r(His-V5)/NcPI-S and r(His-V5)/NcPI-Smut probed with anti-V5. (C) Activity assay of recombinant NcPI-S/NcPI-Smut. Increasing amounts of r(His-V5)/NcPI-S and r(His-V5)/NcPI-Smut were incubated with subtilisin A of *Bacillus lechiniformis*. Residual hydrolytic activity (R.H.A) was measured through substrate conversion.

The Kazal serine protease inhibitor NcPI-S is a 79 amino acid peptide with a theoretical mw of 6 kDa (Fig. 1) [33]. We first expressed NcPI-S in bacteria as a recombinant NcPI-S tagged with V5 and 6xHis (r(His-V5)/NcPI-S) (Fig. 1A). Western blot experiments using a monoclonal Ab recognizing anti-V5 revealed the presence of a predominant band of ∼17 kDa in our preparation (Fig. 1B, right) which is slightly bigger than the predicted 10.7 kDa of the r(His-V5)/NcPI-S. This is consistent with previous studies showing that NcPI-S migrates slower on Tris-tricine SDS-PAGE gels [34] and even slower on Tris SDS-PAGE gels [33,34]. The inhibitor r(His-V5)/NcPI-S was tested against the *Bl*-subA using previously reported enzymatic assays [33,34] (Fig. 1B, left) indicating that the N-amino terminal extension does not interfere with the inhibitory activity of NcPI-S.

In parallel, we used bacterial expression to validate an NcPI-S mutant variant. Although the inhibitory specificity of Kazals is primarily dictated by the identity of the amino acid at the P1 position, other amino acid interactions between the Reactive Site Loop (RSL) and the substrate binding cavities may equally influence the binding of Kazals to proteases [28]. As previously shown, mutagenesis of the P1 Met to Ala of NcPI-S does not affect its inhibitory activity [34]. Thus, we decided to change a significant proportion of the (RSL). To achieve this, we have substituted the NcPI-S’s P2-P4’ site with the sequence FASGKR (NcPI-Smut; Fig. 1A). This sequence corresponds to the non-prime site (P6-P1) of a cleavage site processed by the West Nile Virus (WNV) trypsin-like serine protease [54]. Our hypothesis was to test the inhibitory profile of a serine protease recognition site not present in *P*. *berghei* and *T*. *gondii*. The recombinant mutant (His-V5)/NcPI-Smut, (r(His-V5)/NcPI-Smut), showed a substantial mobility shift in respect to the r(His-V5)/NcPI-S, although the calculated size difference was only 0.2 kDa (Fig. 1B). More importantly the NcPI-S mutant variant failed to inhibit *Bl*-subA in comparison with the r(His-V5)/NcPI-S (Fig. 1C). At a high concentration of r(His-V5)/NcPI-Smut (750 fmol) a low degree of inhibition was detected, although we never detected more than 20% inhibition, compared to the complete inhibition of r(His-V5)/NcPI-S.

### Ectopic expression of NcPI-S in *Toxoplasma gondii* tachyzoites

**Fig 2:**
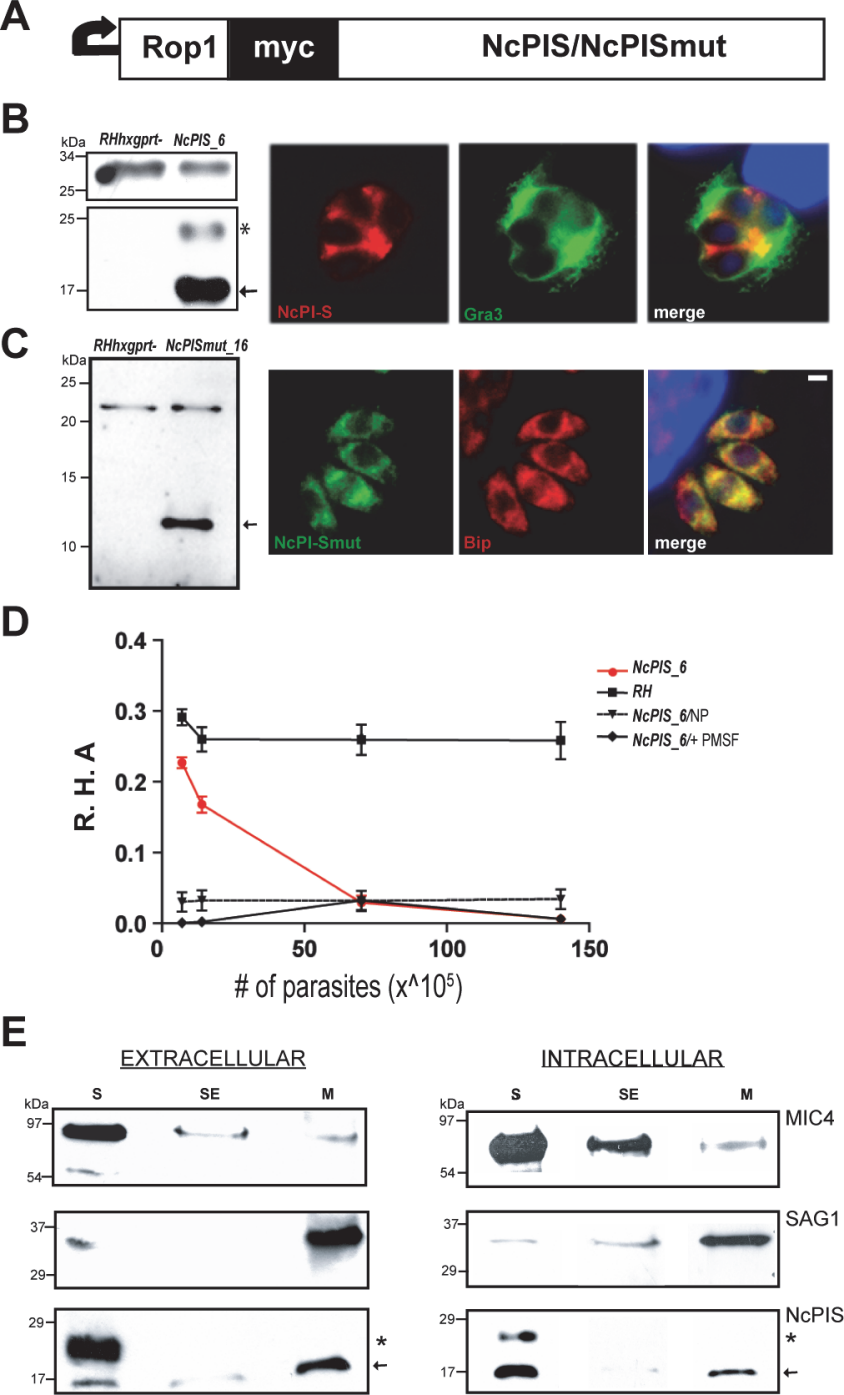
NcPI-S expression in Toxoplasma tachyzoites. (A) *Gra1/ssmycNcPIS* WT or mutant construct is shown indicating the signal sequence of ROP1, the myc-tag and the NcPIS variants. (B) *(left)* Western blot analysis of lysates derived from extracellular parasites of transgenic *NcPIs_6* and RH*hxgprt*–strains. An arrow marks the predominant monomeric form of myc-tagged NcPI-S inhibitor (bottom), while asterisk indicates the additional NcPIS dimeric form. SAG1 protein (top) was used as loading control. *(Right)* Localization of NcPIS (red) and the DG protein GRA3 (green) in intracellular parasites, 24 h p.i. Nuclei stained with DAPI (blue). Scale bars, 1μm. (C) *(left)* Western blot analysis of lysates derived from extracellular parasites of transgenic *NcPIsmut_16 (arrow)* and RH*hxgprt*- strains. GRA1 protein (top) was used as loading control. *(right)* Localization of mutant NcPI-S (green) and the chaperonin Bip protein (red) in intracellular parasites, 24 h p.i. Nuclei stained with DAPI (blue). Scale bars, 1μm. (D) Inhibitory activity of tachyzoite lysates against *Bacillus lechiniformis* (*Bl*) subtilisin. Tachyzoite lysates prepared from *RH* and *NcPIS_6* were incubated with *Bl* subtilisin in the presence of 0.15 mM CBZ-Gly-Gly-Leu-*p*NA substrate. Residual hydrolytic activity (R.H.A) was measured. No activity was detected when lysates were incubated with the substrate alone (*NcPIs_6*/NP) or in reactions in which lysates were incubated with the *Bl* subtilisin in the presence of 2mM PMSF (*NcPIS_6/+PMSF)*. Error bars represent the means ±SEM. (n = 2 experiments per strain, representative of triplicate samples). (E) Distribution of NcPI-S in soluble (S), membrane salt-eluted (SE) and membrane (M) fractions derived from either extracellular *(left)* or intracellular *(right)* parasites. The monomeric and dimeric forms of NcPIS (bottom) are marked with an arrow and asterisk, respectively. MIC4 micronemal protein (upper) and GPI anchored SAG1 (middle) were used as indicators of soluble and membrane associated proteins respectively.

As a proof of concept that ectopic expression of a PPI can affect an aspect of parasite infection, we expressed NcPI-S and the inactive variant NcPI-Smut in *Toxoplasma* tachyzoites. NcPI-S and NcPI-Smut were expressed as amino-terminal fusions of the PPI by replacing its own signal peptide with the ROP1 signal sequence [55] followed by a myc epitope tag under the control of the constitutive *Gra1* gene promoter [56] (Fig. 2A). Transfection of the recombinant plasmids (*pGra1/ssmycNcPIS* or *pGra1/ssmycNcPISmut*) in the RHΔhxgprt recipient strain [35] and clonal selection resulted in several stable clones in both cases. One clone in each case, *NcPIS_6* or *NcPISmut_16* respectively, was selected for further studies.

Western blot analysis of *NcPIS_6* tachyzoite extracts identified a predominant (myc)/NcPI-S band of ∼17 kDa (Fig. 2B left, arrow). An additional fainter band at ∼24 kDa, possibly corresponding to a (myc)/NcPI-S dimer, as has been previously described [34], was also apparent (Fig. 2B, asterisk). The (myc)/NcPI-Smut variant was detected as a single faster migrating band than (myc)/NcPI-S (Fig. 2C) suggesting that the NcPI-Smut dimer might be below the threshold of detection.

Immunofluorescence analysis (IFA) was performed on tachyzoites using a mAb recognizing the myc epitope. These experiments revealed that (myc)/NcPI-S was secreted into the parasitophorous vacuole (PV) when intracellular *NcPIS_6* tachyzoites were labeled (Fig. 2B, right and S1B Fig.), while in extracellular tachyzoites (myc)/NcPI-S exhibited a punctate staining pattern (S1C Fig.). In contrast, no signal was detected in the intravacuolar space of the intracellular growing *NcPISmut_16* tachyzoites. Instead, IFA revealed retention of (myc)/NcPI-Smut within the tachyzoites and specifically within the ER, which in tachyzoites is expanded around the cell nucleus being more prominent posterior to the nucleus. The increased ER retention of (myc)/NcPI-Smut was indicated by its co-localization with the ER-resident chaperone protein BiP (Fig. 2C, right).

In order to verify that the myc-tagged NcPI-S retains its inhibitory potential we used the *Bl*-SubA enzymatic assay to test extracts derived from either WT or *NcPIS_6* extracellular tachyzoites. Only extracts derived from *NcPIS_6* tachyzoites inhibited the *Bl*-SubA activity and moreover this inhibition was directly proportional to the number of parasites that were used to prepare the extracts (Fig. 2D).

Fractionation of extracts derived from extracellular tachyzoites (isolated from infected, spontaneously-lysed cells) or from intracellular tachyzoites (isolated from vacuoles released by force from infected cells 24 h post infection), revealed abundant presence of (myc)NcPI-S in the soluble fraction (S) in both cases (Fig. 2E, lower panels). Association of the (myc)NcPI-S with the membrane pellet (M), (insoluble after treatment with Na_2_CO_3_ that removes membrane associated proteins), was also observed but at a much lower degree (Fig. 2E, lower panels). Importantly, both the ∼17 kDa (arrow) and the potential ∼24 kDa dimer (asterisk) of the (myc)NcPI-S were detected in the S fractions of tachyzoites as has been previously observed [34].

### Expression of NcPI-S in *T*. *gondii* tachyzoites impairs parasite replication

**Fig 3:**
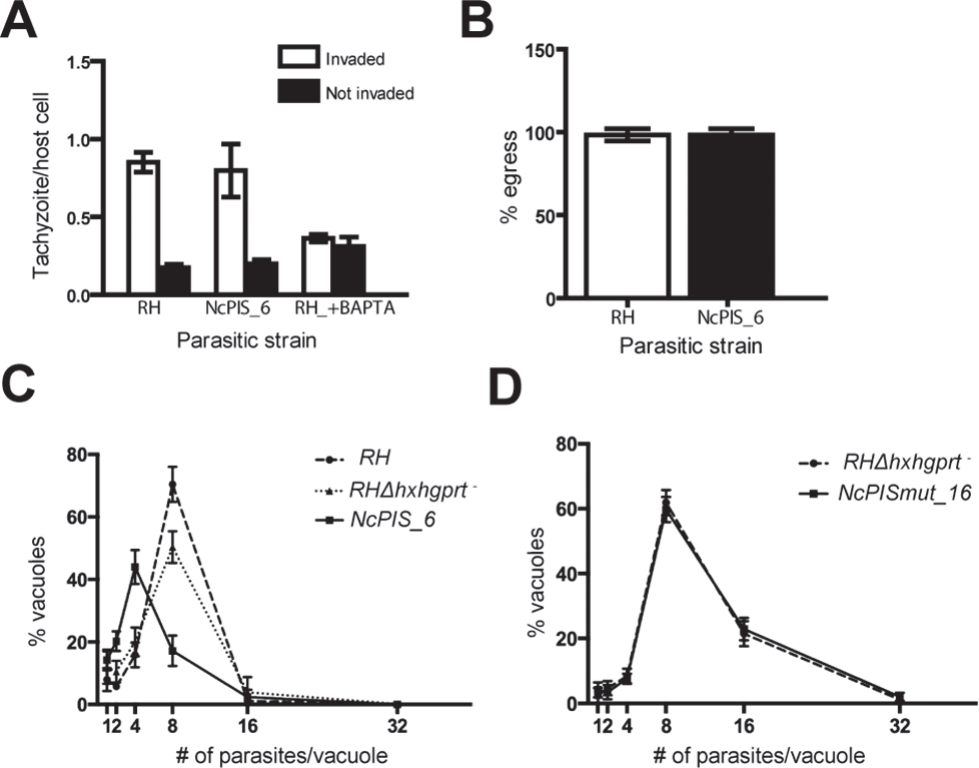
Phenotypic characterization of NcPIS_6 strain. (A) Invasion competence of NcPIS_6 clone was similar to the RH strain. BAPTA-AM treated parasites, which do not invade cells, were included as a control. Error bars represent SEM (n = 3, each experiment was performed in duplicate and ten random fields were screened blindly in each sample). (B) Percentage of lysed vacuoles of NcPIS_6 versus RH after inducing egress with calcium ionophore A23187. Intracellular replicating tachyzoites (36–40 h p.i.) were treated with 1 μM A23187 or DMSO (solvent control) for 5min. Percentage of induced egress from host cells was determined. Error bars, SEM (n = 3, each experiment was performed in duplicate and, 10 fields were blindly screened per sample). (C) Replication assay of NcPIS_6, RH and RHΔhxgprt^-^ (parental) and (D) NcPISmut_16 and RHΔhxgprt^-^. Following a 24 h interval of intracellular replication, vacuoles from all the above strains were scored according to their parasite context. Error bars, SEM (n = 3, representative of duplicate samples); P<0.001, Student’s t-test.

To study the effect of expressing NcPI-S and its mutated form in *T*. *gondii* tachyzoites we performed phenotypic characterization. *NcPIS_6* tachyzoites were compared to WT tachyzoites of the RH and the parental RHΔhxgprt strains using well-established invasion [47], egress [48] and growth assays. Neither invasion (Fig. 3A) nor egress assays (Fig. 3B) revealed any significant phenotypic deviation of *NcPIS_6* from the WT control RH strain (Fig. 3A and 3B). To assess growth we carried out replication assays [57] where equal numbers of tachyzoites from *NcPIS_6* and WT control strains (RH and RHΔhxgprt), were used to infect HFF cells (Fig. 3C). At 24 h p.i., vacuoles derived from control strains contained 8 tachyzoites in a percentage ranging between 40–70% of the total vacuoles, indicating 3 cell divisions as was expected. In contrast, only ∼17% of *NcPIS_6* vacuoles contained 8 tachyzoites (Fig. 3C), indicating impaired replication. A clone (*NcPIS_8*), derived from a second independent transformation experiment, showed similar growth kinetics to that of the *NcPIS_6* (S1A Fig.), verifying that a replication defect of *T*. *gondii* tachyzoites is reproducibly associated with the expression of (myc)NcPI-S. In contrast *NcPISmut_16* tachyzoites showed the same growth dynamics as the parental RHΔhxgprt strain (Fig. 3D). When combined, these results suggest that active NcPI-S is expressed in tachyzoites, and interfere with proliferation of the parasite, while the mutated form has no effect on parasite growth.

### Ectopic expression of NcPI-S and NcPI-Smut in *Plasmodium berghei* zygotes/ookinetes

**Fig 4:**
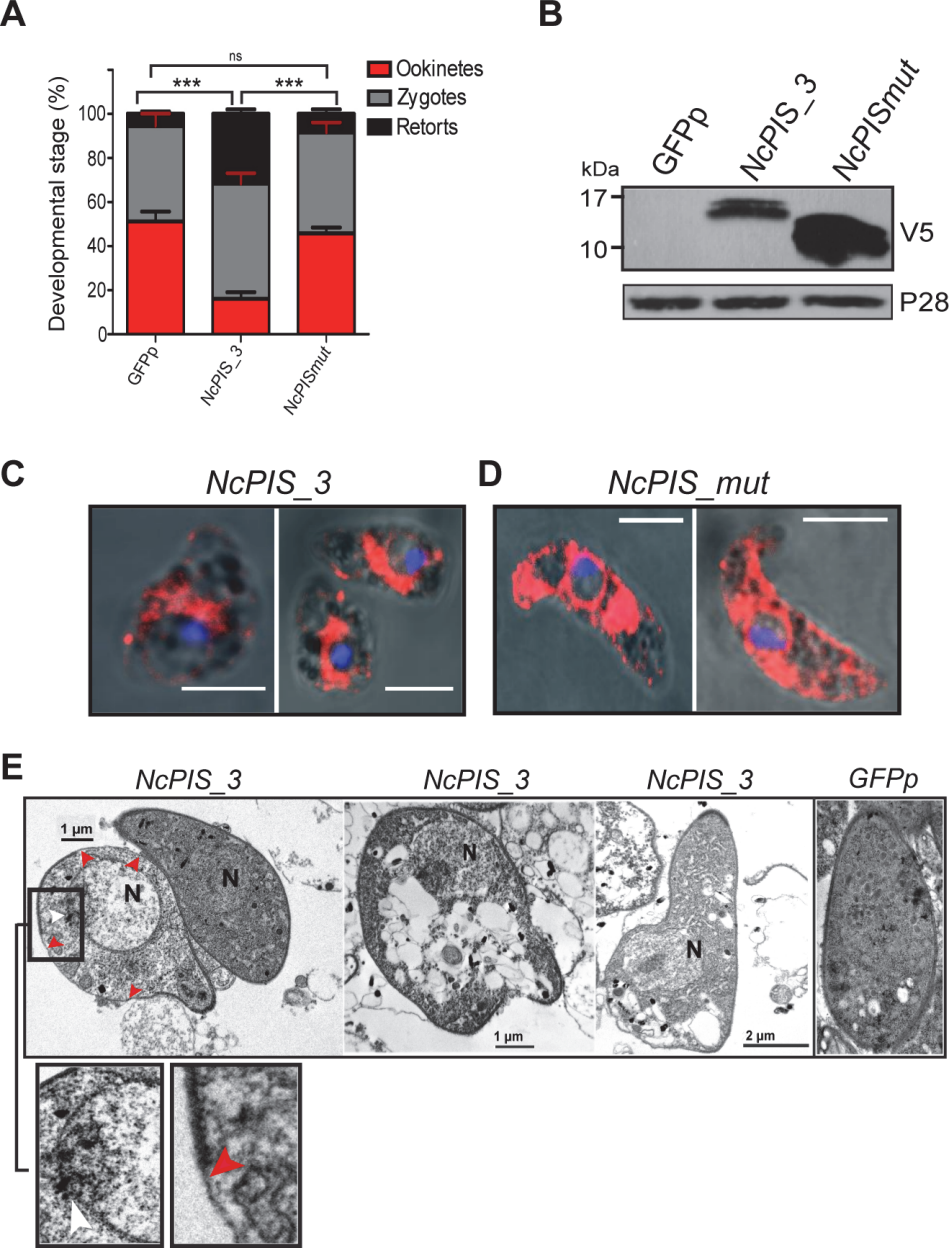
Developmentally arrested NcPIS_3 zygotes/ookinetes show gross abnormalities. (A) Ookinete conversion in *NcPIS_3* and *NcPISmut* parasites versus *GFPp* (WT). The percentage of ookinetes formed in *NcPIS_3* was significantly lower compared to WT and *NcPISmut* parasites. Ookinetes, macrogametes and zygotes were counted after labeling with an antibody recognizing the Pbs21 surface antigen. Error bars represent ± SEM, n = 3. (B) Whole protein extracts from ∼1x10^6^ ookinetes derived from *in vitro* cultured *NcPIS_3*, *NcPISmut* or *GFPp* (WT) parasites were examined for the presence of NcPI-S or NcPI-Smut respectively using anti-V5 mAb. In *NcPIS_3* extracts a predominant ∼17 kDa band was detected while a band of ∼14 kDa was detected in extracts of *NcPISmut*. P28 was used as a loading control. (C-D) Subcellular distribution of NcPI-S and NcPI-Smut in ookinetes of transgenic *P*. *berghei* parasites. Confocal microscopy using anti-V5 MAb (red) shows the different localization of *NcPIS_3* and *NcPISmut* in parasites. In *NcPISmut* there is dispersed and perinuclear staining pattern compared to the *NcPIS_3* where expression is in close proximity to the nucleus. Nucleus is stained with TO-PRO-3 (blue). Scale bar 5 μM. (E) Transmission electron micrographs (TEM) of arrested *NcPIS_3*, zygotes/ookinetes show gross abnormalities. The endomembrane system is dilated. Left image shows an arrested parasite with discontinuities in the inner membrane (red arrowheads) while release of content from the nucleus (N) to the cytoplasm is manifested (white arrowhead).

The results from the analysis of *T*. *gondii* encouraged us to test similar constructs in *Plasmodium*, to determine the effect of NcPI-S in these parasites. To this end, recombinant *P*. *berghei* parasites were produced using standard genetic transfection protocols. In brief, the constructs were designed to express V5 epitope tagged NcPI-S and NcPI-Smut fused to the *pbsub2* signal peptide sequence [58], to ensure correct sorting in the secretory apparatus. The engineered open reading frames (ORFs) were placed under the control of the 5’ and 3’ UTRs of *ctrp* and *p28* genes, respectively, which drive expression in the late zygote and ookinete stages [40]. The construct was integrated in the *c-ssu/d-ssu* locus of the *GFP507cl1* (GFPp) strain, constitutively expressing GFP [37] (S2A Fig.). We chose to restrict expression of NcPI-S and its inactive variant NcPI-Smut to the zygote/ookinete stages in *P*. *berghei* to avoid any toxic effects in the asexual blood stages, in which transfection is carried out. Clonal lines were established and one clone from each transfection (*NcPIS_3* and *NcPISmut*) was selected for further analysis after genotypic characterization (S2B and S2C Fig.). As expected asexual growth and gametocytogenesis were both unaffected in *NcPIS_3* and *NcPISmut* parasites (data not shown). However, when ookinete conversion was scored in *in vitro* cultures of the two strains, we found that the majority of *NcPIS_3* parasites were developmentally arrested after zygote formation. However, ∼13% normal ookinetes were detected in each experiment. In contrast, the *in vitro* cultures of the *NcPISmut* strain produced ookinetes at numbers comparable to the parental *GFPp* strain (Fig. 4A).

Expression of (V5)/NcPI-S and (V5)/NcPI-Smut, was confirmed by Western blotting analysis of *in vitro* cultured ookinetes. Consistent with the size of the bacterially expressed inhibitors, the anti-V5 mAb detected a predominant ∼17 kDa band in extracts of the *NcPIS_3* parasites, while a ∼13 kDa band was detected in extracts of the *NcPISmut* strain (Fig. 4B). IFAs using the V5 mAb revealed that in the developmentally arrested cells of the *NcPIS_3* strain the PPI was localized in an area proximal to the nucleus (Fig. 4C, *NcPIS_3*). In *NcPISmut* ookinetes the inhibitor was detected throughout the entire cytosol including a perinuclear signal possibly coinciding with the ER, which in zygotes/ookinetes is composed of only a few tight stacks encircling the nucleus (Fig. 4D, *NcPISmut*). To further examine the potential localization of NcPI-S and NcPI-Smut we have performed double staining with the ER chaperone BiP (S3 Fig.). In the case of *NcPIS_3* parasites, some partial colocalization is seen. In these retort forms though and based on the TEM experiments, the ER might be severely rearranged. In the *NcPISmut* strain there is almost complete colocalization of NcPI-Smut with BiP as has been also the case in intracellular *T*. *gondii* parasites expressing NcPI-Smut (Fig. 2C).

Transmission Electron Microscopy (TEM) was used to further examine the morphological defects of *NcPIS_3* developmentally arrested ookinetes. We detected a pronounced dilation of the endomembrane system with electron dense aggregates being observed (Fig. 4E, middle and right, S4A Fig.). Importantly, this dilation appeared also polarized, in accordance with the (V5)/NcPI-S localization pattern (compare Fig. 4C left and Fig. 4E middle). Other developmentally arrested ookinetes showed a progressive degeneration (Fig. 4E, left) manifesting a range of ultrastructural defects such as incomplete formation of the inner membrane (Fig. 4E, red arrowheads). In some extreme cases of terminally degenerated ookinetes the whole cytoplasm was replaced with vacuoles of different sizes (Fig. 4E, middle, S4B Fig.). In conclusion, our analysis revealed that the expression of (V5)/NcPI-S during zygote to ookinete transition resulted in a prompt and almost total block in development.

### NcPIS_3 parasites are impaired in oocyst formation

**Fig 5:**
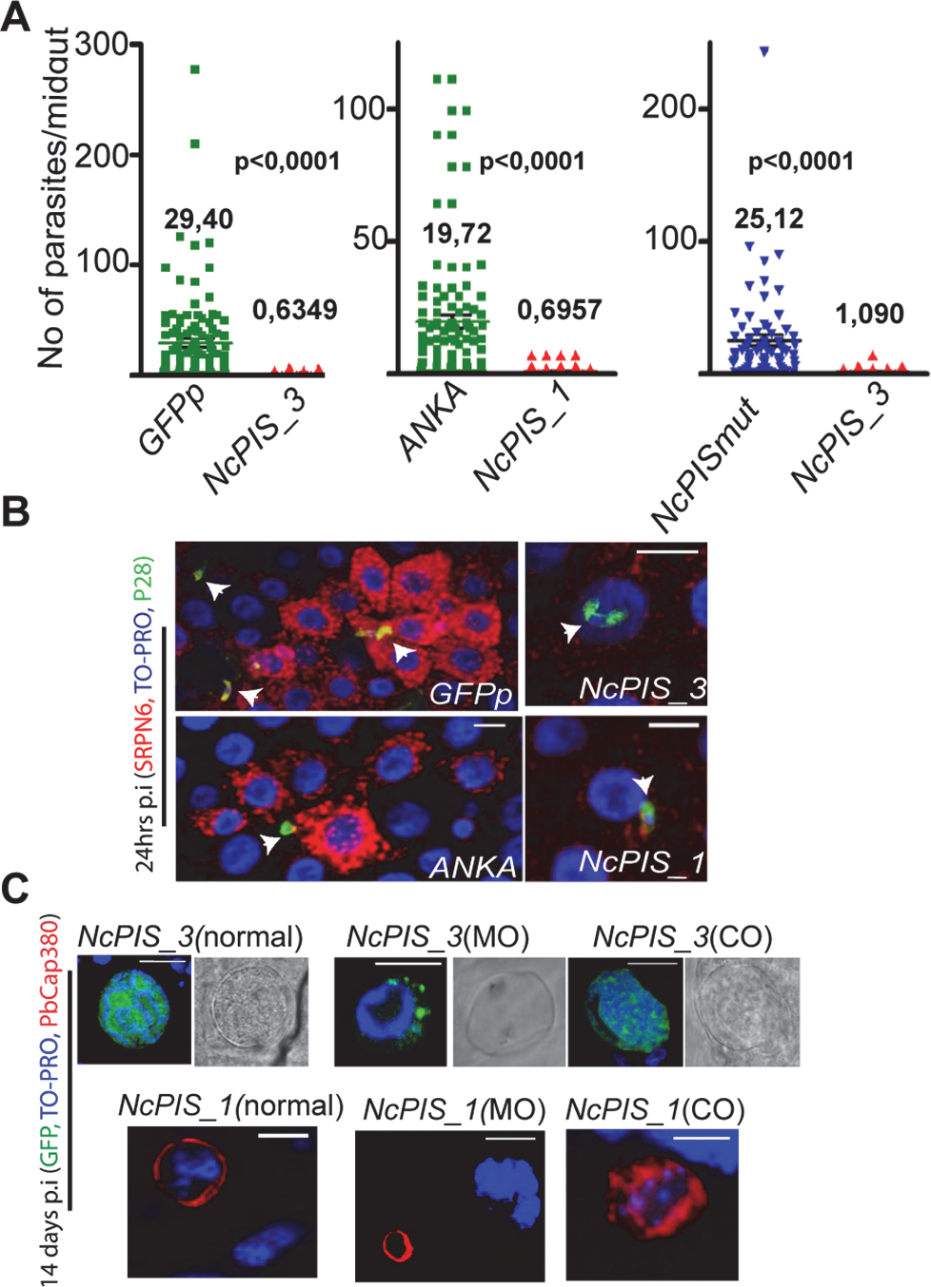
NcPIS_3 *Plasmodium* ookinetes fail to produce normal oocysts. (A) Oocyst formation of NcPI-S expressing clones (*NcPIS_3* and *NcPIS_1*) compared to that of the parental WT strains (*GFPp* and *ANKA* respectively) and of *NcPISmut* compared to *NcPIS_3* 7–10 days post infection. Four sets of paired infection experiments were included in the case of *NcPIS_3*/*GFPp*, two in the case of *NcPISmut*/*NcPIS_3* and three in the case of *NcPIS_1*/ANKA 2.34 paired infections. Error bars; SEM; P<0.001, Mann-Whitney test, in all three cases. (B) Confocal sections of representative mosquito midgut epithelial sheets infected with *GFPp*, *NcPIS_3*, ANKA or *NcPIS_1* parasites, and stained with antibodies against the mosquito serpin6 (SRPN6) (red) and the ookinete surface protein P28 (green). Nuclei are stained with TO-PRO 3 (blue). Arrows indicate the position of ookinetes. *NcPIS_1* and *3* ookinetes fail to induce an epithelial response, which in contrast is revealed in the case of ANKA and *GFPp* infected midguts by SRPN6 specific antibody. Scalebar 10μM. (C) Confocal microscopy of mature oocysts. The two types of *NcPIS_3* oocysts (upper panel) are shown alongside the single oocyst that showed a normal morphology 14 days post infection. Main oocyst morphology includes: Normal sized oocysts with diffused nuclear material and a collapsed capsule (CO) and oocysts arrested in an early developmental stage (minute oocysts: MO). Green corresponds to GFP expression; nuclei are stained with TO-PRO 3. (Lower panel) *NcPIS_1* abnormal oocysts labeled with the antibody recognizing the oocyst capsule protein PbCap380. Scale bar 20μM.

To assess the ability of *NcPIS* expressing parasites to transmit through mosquitoes we fed *A*. *gambiae* female mosquitoes on mice infected with the strain *NcPIS_3* and the clone *NcPIS_1* derived from an independent transfection using the ANKA reference strain as a recipient. The result revealed a 97–98% reduction of in oocyst numbers in mosquitoes infected with either *NcPIS_3* or *NcPIS_1* parasites, compared to infection with the parental strains (Fig. 5A, left and middle respectively) or in comparison to the *NcPISmut* strain (Fig. 5A, right). Midguts were also dissected and stained 24 h after feeding with *NcPIS_3* or *NcPIS_1*. For these IFAs, ookinetes were labeled with the P28 antibody and with an antibody directed against the *A*. *gambiae* serine protease inhibitor SRPN6 (AgSRPN6), which selectively labels invaded cells [59]. Thus we confirmed that a reduced number of ookinetes invaded the midgut epithelium (Fig. 5B). In addition we investigated the effect of NcPI-S on the sporogony of the few surviving *NcPIS_3* and *NcPIS_1* oocysts (Fig. 5C). To determine if these oocysts develop normally, IFA analysis was performed with the oocyst capsule protein PbCap380 [53] and with nuclear staining for the presence of sporozoites. From a total of 33 *NcPIS_3* oocysts recorded in three independent infection experiments only a single oocyst showed a normal morphology (budded sporozoites), hence bite back experiments were not performed. The remaining oocysts fall into two categories: smaller than the expected size, suggesting arrested growth and nuclear division (minute oocysts, MO), or relatively normal in size with a collapsed capsule containing diffused nuclear material (collapsed oocysts, CO) (Fig. 5C). Similarly, among twelve recorded *NcPIS_1* oocysts, three had a normal morphology, four were MO and five were CO.

### Microneme formation is severely reduced in NcPIS_3 ookinetes

**Fig 6:**
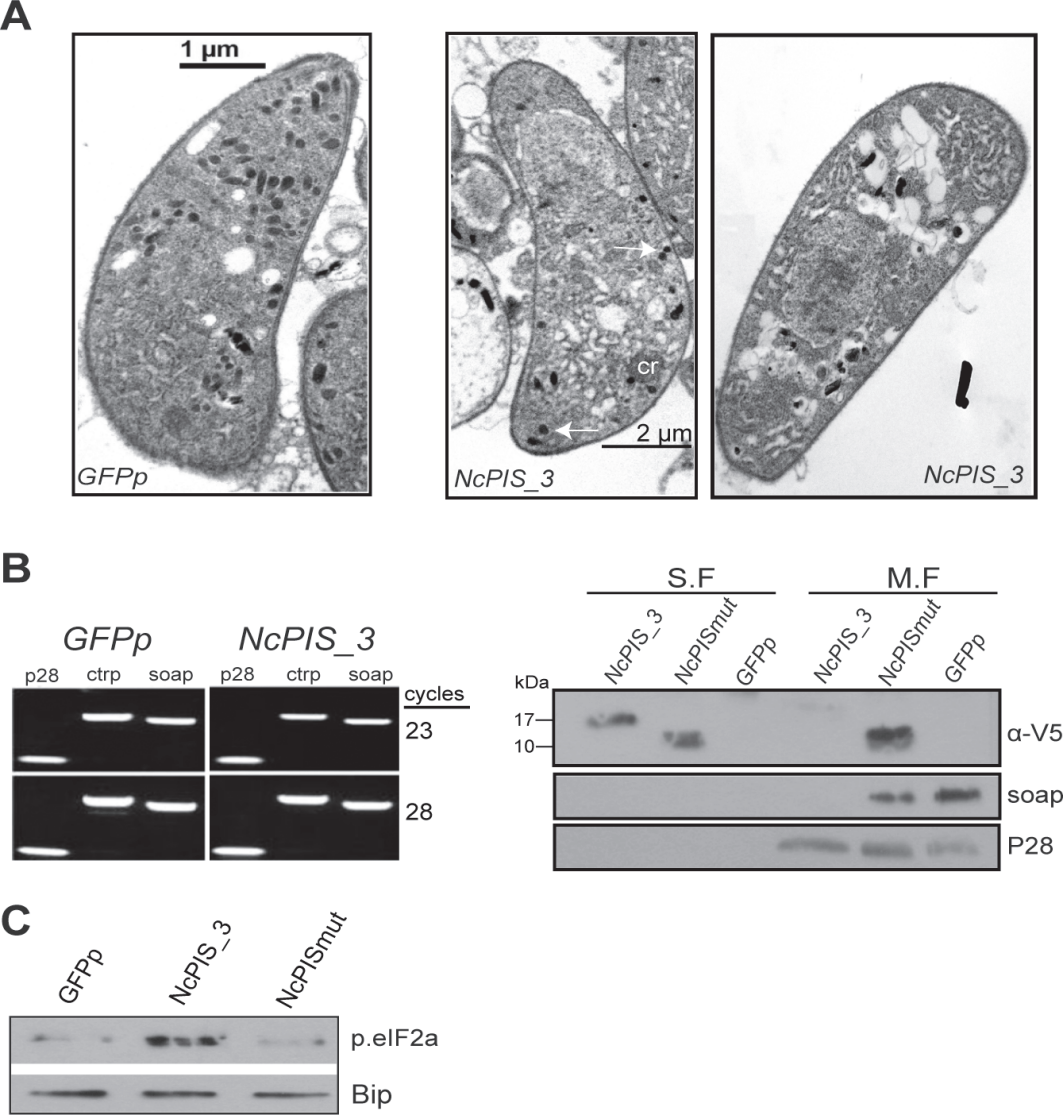
Mature NcPI-S_3 ookinetes contain fewer micronemes. (A) Selected longitudinal sections, across the apical part of wild type (left image) and *NcPIS_3* mature ookinetes (middle and right images). All are surrounded by the characteristic subpellicular inner membrane complex. Scarcity of micronemes (white arrows), is a common feature in *NcPIS_3* ookinetes. The ookinete-specific characteristic structure of crystalloid (cr) is clearly visible. (B) (Left panel) Semi-quantitative RT-PCR analysis of micronemal proteins CTRP and SOAP in *NcPIS_3* and *GFPp* ookinetes. No alteration was observed in transcript levels. *p28* was used as a control. (Right panel) Western analysis of soluble (SF) and membrane (MF) fraction of ookinete extracts derived from *in vitro* cultured *NcPIS_3*, *NcPISmut* and *GFPp* ookinetes, with antibodies against the micronemal protein SOAP. P28 was used as a sample normalization control. No SOAP is detected in the *NcPIS_3* line. (C) Western blot analysis of PbeIF2a phosphorylation. Western blots loaded with lysates from *GFPp*, *NcPIS_3* and *NcPISmut* ookinete cultures were probed with antibodies against phosphorylated eIF2α. *NcPIS_3* parasites show an increase in PbeIF2a phosphorylation. The endoplasmic reticulum (ER) marker, BiP was used as the loading control.

Careful inspection of the TEM pictures revealed that the few mature *NcPIS_3* ookinetes which developed, suffered from ultra-structural malformations including a severe decrease in the number of micronemes (Fig. 6A). To further investigate this we chose to study the micronemal protein SOAP. We first verified that the *soap* transcript levels in the *NcPIS_3* zygotes/ookinetes were comparable to WT (Fig. 6B left). However, Western blot analysis showed that the micronemal protein SOAP [60] was hardly detectable in *NcPIS_3* zygote/ookinete extracts, compared to *GFPp* or *NcPISmut* derived extracts where this protein was readily seen (Fig. 6B right). Furthermore we repeated these experiments using the *NcPIS_1* parasite clone to exclude any effects deriving from the genetic background. The results were identical (S5 Fig.).

In apicomplexans, insults that perturb ER homeostasis result in repression of translation which is mediated by the increased phosphorylation of the eukaryotic Initiation Factor 2a (elF2a) [61,62]. To investigate if such an effect was caused by the expression of NcPI-S we examined the levels of elF2a phosphorylation by using a Phospho-eIF2α (Ser51) antibody, that was previously shown to cross-react with the phosphorylated form of the *P*. *falciparum* eIF2a [63], on Western blots containing extracts from *NcPIS_3*, *NcPISmut* and *GFPp* zygote/ookinete cultures. A significant increase in PbeIF2a phosphorylation levels was revealed in *NcPIS_3* extracts as compared to extracts derived from the *GFPp* and *NcPISmut* (Fig. 6C). Since we did not reveal any significant alteration in the transcript levels of SOAP (as well as of CTRP) in the *NcPIS_3* strain as compared to WT (Fig. 4B), the marked decrease in the SOAP protein levels could probably reflect the impaired translation in *NcPIS_3* parasites.

## Discussion

In this study we investigated the use of serine protease inhibitors of the Kazal type to interfere with *T*. *gondii* tachyzoite egress, invasion and growth and *P*. *berghei* zygote to ookinete transition. We chose NcPI-S, a Kazal inhibitor originating from the related apicomplexan parasite *N*. *caninum*. Firstly, we expressed NcPI-S in *T*. *gondii* in an attempt to validate the PPI in a fast and reliable manner. After initial validation we expanded our studies by designing a strategy chosen to restrict the expression of the inhibitor in the *P*. *berghei* zygote to ookinete transitional stage, a crucial time in the life cycle of malaria species as failure to successfully complete this step blocks transmission. Importantly, this timing of expression was also chosen as it circumvented the possible detrimental effects on viability parasites of the blood stages, in which the genetic transfection takes place.

All three characterized apicomplexan Kazal inhibitors (TgPI-1, TgPI-2 and NcPI-S) are dense granule (DG) constituents. The ectopically expressed NcPI-S in *Toxoplasma* tachyzoites was also trafficked in DGs and secreted in the parasitophorous vacuole, similarly to the endogenously expressed NcPI-S in *N*. *caninum* tachyzoites. Strikingly though, the catalytically dead variant NcPI-Smut was retained within the tachyzoite ER. This could be explained if NcPI-S piggybacks on its potential targets through the secretory pathway, or if RSL mutagenesis disrupted a sorting signal enabling NcPI-S anterograde trafficking. Additional studies will be necessary to distinguish between these possibilities. Interestingly, there are conserved domains in the RSLs prime site (P2’ to P5’) of several Kazal domains including NcPI-S, but not in any of the Kazal domains of TgPI-1 and TgPI-2 inhibitors. The biological role of these though, if any, is yet to be defined.

In *T*. *gondii* the ectopically expressed NcPI-S in *Toxoplasma* tachyzoites was secreted in the parasitophorous vacuole, presumably via the DGs, similar to the endogenously expressed NcPI-S in *N*. *caninum* tachyzoites [34]. Strikingly though, a catalytically dead variant NcPI-Smut was retained within the ER of the tachyzoite. In contrast to the native NcPI-S no dimer was observed for the mutant form. This could be attributed to structural changes in the mutant preventing its efficient dimerization. For their ectopic expression in each case, NcPI-S and NcPI-Smut were fused to the same signal peptide and epitope tag sequences, differing in 6 amino acids within the RSL including the P1-P1’ residues that are critical for the inhibitory activity. Possible reasons firstly for this differential localization of NcPI-S and NcPI-Smut in tachyzoites might be that either NcPI-S is trafficked within the secretory pathway piggybacked on its potential targets, or that RSL mutagenesis disrupted the folding of a sorting signal enabling NcPI-S anterograde trafficking as well as affected the ability of the protein to homodimerize.

Fractionation of extracellular and 24 h intracellular tachyzoites expressing active NcPI-S revealed its presence in the soluble fraction, with the ∼24 kDa dimer being more abundant in the soluble fraction of the extracellular tachyzoites. In the study of Morris *et al*. [34] the endogenous NcPI-S in *N*. *caninum* was also shown to form a dimer, with anomalous migration (∼20 kDa) in extracellular tachyzoite extracts, which is in accordance with our observations. Furthermore, our results imply that NcPI-S dimerization depends on the subcellular environment (dense granules versus vacuole). However, presently we do not know to which degree dimerization influences the NcPI-S inhibitory capacity. Interestingly, phenotypic characterization of NcPI-S and NcPI-Smut strains revealed that while the presence of NcPI-Smut in *T*. *gondii* was not detrimental for tachyzoites, ectopic expression of NcPI-S significantly affected their intracellular growth. Parasite replication was impaired, suggesting the possibility that NcPI-S interfered with a proteolytic event during parasite replication.

Furthermore, ectopic expression of NcPI-S in *P*. *berghei* zygotes blocked their differentiation to ookinetes. Consequently, oocyst production was severely impaired and the few surviving oocysts were defective in sporulation. In contrast, ectopic expression of the inactive NcPI-Smut did not have any effect on zygote to ookinete differentiation *in vitro*, and on mosquito virulence *in vivo*. In TEM studies the majority of the developmentally arrested ookinetes showed a range of morphological abnormalities indicative of a fatal toxic stress. The few morphologically mature ookinetes also exhibited severe defects with the most prominent being a drastic reduction of the number of micronemes. In line with the reduced number of micronemes, the protein level of the micronemal protein SOAP was significantly reduced even though the RNA levels of this molecule were unaffected. Additional studies, showed elF2a increased phosphorylation in *NcPIS_3* zygotes/ookinetes that is indicative of translational repression. Studies in other organisms (yeast, *Toxoplasma*), have shown that apart from the reduced global protein synthesis due to the eIF2a phosphorylation, preferential translation of certain proteins helps the cells to cope with the elevated stress [64]. If such is the case also in *Plasmodium*, it might not be surprising that SOAP levels are decreased, in contrast to the BiP levels, (major ER chaperone in many organisms) which remain unaffected. It is noteworthy that inhibition of translation by the use of cycloheximide results in a block in zygote to ookinete development (Siden-Kiamos and Deligianni, unpublished).

Similarly to our study in *T*. *gondii* it was recently shown that elimination of one of the Kazal inhibitors expressed in *Toxoplasma*, TgPI-1 (Δ*Tg*PI1), results in higher parasite burden *in vivo* suggesting that the endogenously expressed Kazal inhibitors also have a negative effect on growth of the parasite [65]. Furthermore, Δ*Tg*PI1 parasites exhibited additionally increased bradyzoite gene expression and more efficient *in vitro* differentiation after stress of the tachyzoites in low CO_2_ and alkaline pH [65]. Even though the target protease of TgPI-1 hasn’t been identified the construction of the Δ*Tg*PI1 line led to the conclusion that PPIs can affect *T*. *gondii* virulence and bradyzoite differentiation. The removal of a negative PPI regulator of proteases in Δ*Tg*PI1 parasites emphasizes the importance of the tight regulation of proteases in apicomplexans. In contrast to TgPI-1, the function of NcPI-S in *N*. *caninum* is yet to be defined. The notion, though, is that it might suppress proteolytic activity to protect resident PV proteins from non-regulated proteolysis [34]. Similarly ectopic expression of active NcPI-S in *T*.*gondii*, shows that addition of an exogenous PPI represents an insult against the balanced regulation of protease activity, which is manifested by defects in parasite replication. More importantly, the impaired oocyst development observed in *P*. *berghei*, the genome of which does not encode a serine PPI, indicates that insults to the regulation of proteolysis cannot be tolerated in a system that has evolved to regulate proteolysis by PPI-independent mechanisms. Since our efforts to generate transgenic *P*. *berghei* parasites expressing NcPI-S under a constitutive promoter (eIF2a and hsp70) have so far been unsuccessful (Koussis and Loukeris, unpublished), we assume that expression of NcPI-S in blood stages is probably detrimental for the parasite.

A key question though that needs to be addressed in future studies is the identity of the target protease(s) of NcPI-S. The effects of NcPI-S in *T*. *gondii* tachyzoites and in *P*. *berghei* zygotes/ookinetes are theoretically mediated through the disturbance or the complete inhibition of processes involving subtilisin-like or rhomboid proteases. An impaired tachyzoite replication rate was previously reported as the main phenotypic manifestation conditionally depleting a micronemal *Toxoplasma* rhomboid TgROM1, which shows residual Golgi localization in tachyzoites [66]. Our notion though is that studies should also focus on other classes of molecules. In *Plasmodium spp* and *Toxoplasma* genomes, proteins with a rhomboid fold have been also identified (Derlins (Ders)). These are incapable of proteolysis but it is believed that they interact with their client proteins in ways similar to the rhomboid proteases [67]. *Plasmodium spp*. and *T*. *gondii* genomes encode for a yeast Der1p orthologue, hDer1-1, which is likely part of the ER Associated Degradation (ERAD) machinery, and a second paralogue sDer1-1, which is part of a duplicated ERAD-like translocon complex placed in the apicoplast [68]. This duplicated ERAD-like translocon is possibly responsible for the transfer of apicoplast proteins across the apicoplast plasma membranes. It would therefore be challenging to attempt silencing of Ders at the zygote stage (by promoter swap) and investigate their effect on zygote to ookinete transition and on the structural integrity of the zygote/ookinete, and compare it with the NcPI-S line.

Apart from genetic studies, *in vitro* biochemical studies could also be implemented to find interacting partners. The recombinantly expressed NcPI-S and its mutant form can be used for pull down assays as has been previously done with a subtilisin Kazal inhibitor expressed by the oomycete pathogen *Phytophthora infestans* [69]. Unfortunately, antibodies for serine proteases in Apicomplexa are not available in many cases and therefore one possibility would be a combination of pull down assays and a comparison of the whole proteome profiles of WT parasites and those that express exogenous PPIs. Such an approach could help to delineate the role of exogenous PPI in both parasites and identify the target proteases of NcPI-S.

The results from this study support our initial hypothesis that NcPI-S (and potentially other subtilisin specific Kazal inhibitors) may be used as a tool to interfere with the physiology of the secretory apparatus of apicomplexan parasites, and in this way to manipulate their viability and growth. With our study we exploited *T*. *gondii* parasites as a system for biochemical and phenotypical validation of the chosen PPI. More importantly, since our main focus is *P*. *berghei* mosquito’s stages our interest is to develop tools that could lead to novel transmission blocking strategies. Previously, studies have demonstrated the antiparasitic activity of antiretroviral protease inhibitors against *P*. *falciparum* [70], *Leishmania* [71] and *T*. *gondii* [72,73]. In addition a native Kunitz-type serine protease inhibitor was recently proven to affect viability of *T*. *cruzi* [74] and *Leishmania* [75]. The unique aspect of our study is that we chose to create transgenic parasite lines with the PPI. We anticipate that by controlling the stage and the timing for NcPI-S expression, as well as NcPI-S’s subcellular localization it may be possible to generate parasitic strains growth attenuated in the vertebrate host, and at the same time incapable of transmission.

## Supporting Information

S1 Fig Phenotypic analysis of *NcPIS* in *T*. *gondii*. **A.** Growth rate of *NcPIs_8* clone compared to that of RH. After 24 hrs of intracellular replication, vacuoles from the above strains were scored according to their parasite context (1, 2, 4, 8, 16 and 32 parasites/vacuole). Error bars represent the means ±S.E (n = 3 experiments per strain, duplicated samples/experiment); statistical significance was determined using t-test (*p<0*.*001)*. **B.** 24hrs *NcPIS_8* intracellular tachyzoites, stained with a-myc antibody that detects NcPI-S (red) secreted into the parasitophorous vacuole partly co-localizing with the dense granule specific protein GRA3 (green). Fluorescent images were collected at 100X with a Zeiss Axioscope2plus microscope equipped with a CCD camera. Nuclei stained with DAPI (blue). Scale bars, 10μm. **C.** Extracellular freshly lysed parasites 48 hrs p.i. were stained with-anti myc (red) and anti-ROM4 (Rhomboid protease 4) (green) antibodies. Nuclei were stained with DAPI (blue). Scale bars, 1μm.
(PDF)

S2 Fig Strategy for the generation of transgenic NcPI-S expressing *P*. *berghei* parasites. **A**. Schematic representation of the gene targeting strategy used for integrating NcPIS or NcPIsmut in the endogenous cssu-rrna locus. Double arrowhead line indicates the expected PCR product for an intact locus. Primers used for diagnostic PCR (L665, L260, L270 and L740) are indicated. **B.** PFIGE (Pulse-Field Inverted Gel Electrophoresis) analysis of transfected parasites. (i) DNA from transfected parasites run on 1% agarose gel, showing chromosomes separated according to their molecular size; Chromosomes 5/6 (which can’t be separated from each other) and 7 are indicated by arrows. (ii) The blot from the same gel hybridized with the 3’ UTR sequences of *Pbdhfr/ts*. The probe detects the 3’ UTR of the endogenous *dhfr* gene on chromosome 7, and the 3’ UTR of the selectable marker indicating correct integration on chromosome 5. **C.** PCR genotypic analysis of the derived transgenic clones. Upper panel: Lane 3 corresponds to *NcPIS_3* clone. The primer pair a (L665/L740) amplifies the integration diagnostic 2.1kb band from genomic DNA isolated from all clones. Failure to amplify a 3kb band using the primer pair c (L270/L740) from gDNA of the two clones indicates that integration took place at the *cssu-rrna* locus, while presence of a 3kb band amplified by the primer set b (L260/L740) in WT control and the two NcPI-S expressing clones indicates the integrity of *dssu-rrna* locus. (Lower panel) The same primer sets were used to confirm integration of *NcPISmut* and *NcPIS_1* in the specified locus.
(PDF)

S3 Fig Localization studies of NcPI-S/NcPI-Smut and Bip in ookinetes. IFA analysis of *NcPI_S3* and *NcPI_Smut* lines, showing expression of the PPI (red) and the ER-chaperone BiP (green) in *P*. *berghei* ookinetes. NcPI-Smut is expressed throughout the cytoplasm and the ER, while NcPI-S has limited colocalisation with BiP. Scale bar: 5 μM.
(TIF)

S4 Fig TEM images of arrested zygote/ookinetes. **A.** Developmentally arrested zygotes exhibiting the characteristic dilation of the endomembrane system alongside extensive vacuolation. **B.** Longitudinal sections of a *sgIV* ookinetes, in which the cytoplasm has been entirely replaced by electron-lucent vacuoles. Some subcellular structures, however, are still identifiable, such as subpellicular microtubules (arrowheads) and a well-structured collar with the characteristic electron-dense depositions surrounding the apical split; notice vacuole excretion from the apical split. A nucleus remnant (N) with a persistent electron dense area is defined by a rough envelope (red arrow). Reduced number of micronemes is also shown in one ookinete (white arrow).
(PDF)

S5 Fig Western analysis of extracts derived from *in vitro* ookinete cultures of *NcPIS_1* clone. Western analysis of extracts derived from *in vitro* ookinete cultures of *NcPIS_1* and the parental *ANKA 15cy1A* (*ANKA*) strain, with V5 Mab (revealing NcPI-S) and antibodies against the micronemal protein SOAP, and the late zygote/ookinete specific P28 protein (sample normalization control). While the levels of P28 are similar between the parental *ANKA* strain and the NcPI-S clone, a reduction in the levels of SOAP is observed in the case of *NcPIS_1* derived extracts.
(PDF)

S1 Table Primers used for DNA construct generation and genotype analysis. (DOCX)
