# Correction: Ectopic Expression of a *Neospora caninum* Kazal Type Inhibitor Triggers Developmental Defects in *Toxoplasma* and *Plasmodium*

**DOI:** 10.1371/journal.pone.0125852

**Published:** 2015-04-23

**Authors:** The PLOS ONE Staff

The following information is missing from the Funding section: “This work was supported by the following grants: WHO V30/181/259, LSHP-CT2004-503578/BioMalPar, AP5243 of the Hellenic General Secretariat of Research and Technology, «Supporting Postdoctoral Researchers- Research project PROTEOMAL LS6-158/AP883» of the Operational Program "Education and Lifelong Learning" (Action’s Beneficiary: General Secretariat for Research and Technology), cofinanced by the European Social Fund (ESF) and the Greek State (to Konstantinos Koussis) and a donation of the Bodosakis Foundation to TGL. RSM was a PhD Fellow of BioMalPar. The funders had no role in study design, data collection and analysis, decision to publish, or preparation of the manuscript.”

The publisher apologizes for the error.
