# Supplementary figures and images for: Ectopic Expression of a Neospora caninum Kazal Type Inhibitor Triggers Developmental Defects in Toxoplasma and Plasmodium
Source: PLoS One. 2015 Mar 24;10(3):e0121379. doi: 10.1371/journal.pone.0121379 (PMC4372514; doi:10.1371/journal.pone.0121379)

A

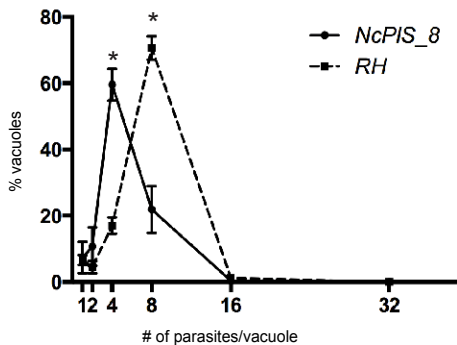

B

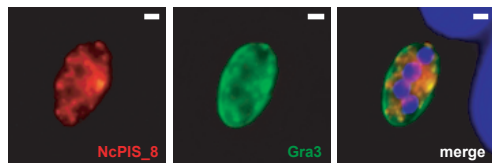

C

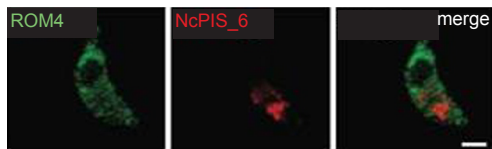

Supplement: S1 Fig — A. Growth rate of NcPIs_8 clone compared to that of RH. After 24 hrs of intracellular replication, vacuoles from the above strains were scored according to their parasite context (1, 2, 4, 8, 16 and 32 parasites/vacuole). Error bars represent the means ±S.E (n = 3 experiments per strain, duplicated samples/experiment); statistical significance was determined using t-test (p<0.001). B. 24hrs NcPIS_8 intracellular tachyzoites, stained with a-myc antibody that detects NcPI-S (red) secreted into the parasitophorous vacuole partly co-localizing with the dense granule specific protein GRA3 (green). Fluorescent images were collected at 100X with a Zeiss Axioscope2plus microscope equipped with a CCD camera. Nuclei stained with DAPI (blue). Scale bars, 10μm. C. Extracellular freshly lysed parasites 48 hrs p.i. were stained with-anti myc (red) and anti-ROM4 (Rhomboid protease 4) (green) antibodies. Nuclei were stained with DAPI (blue). Scale bars, 1μm. (PDF) [file pone.0121379.s001.pdf]

**A**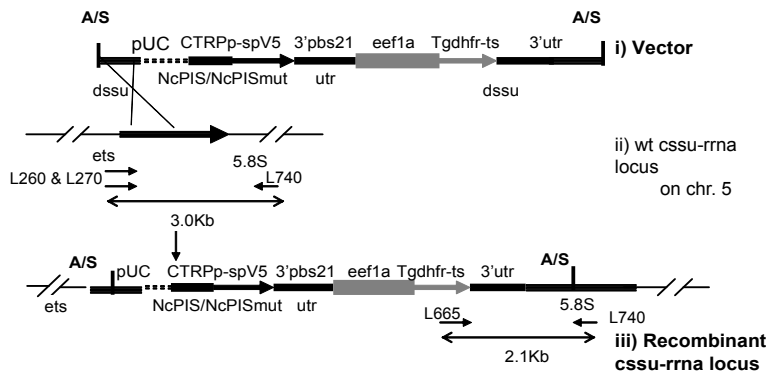**B**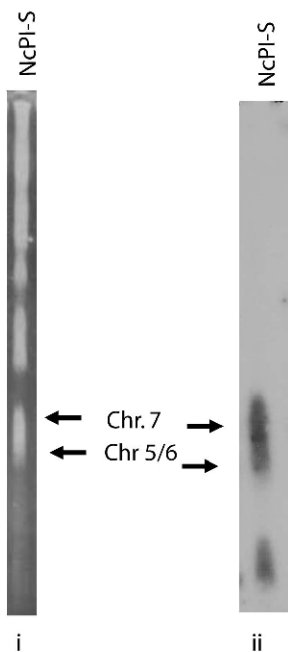**C**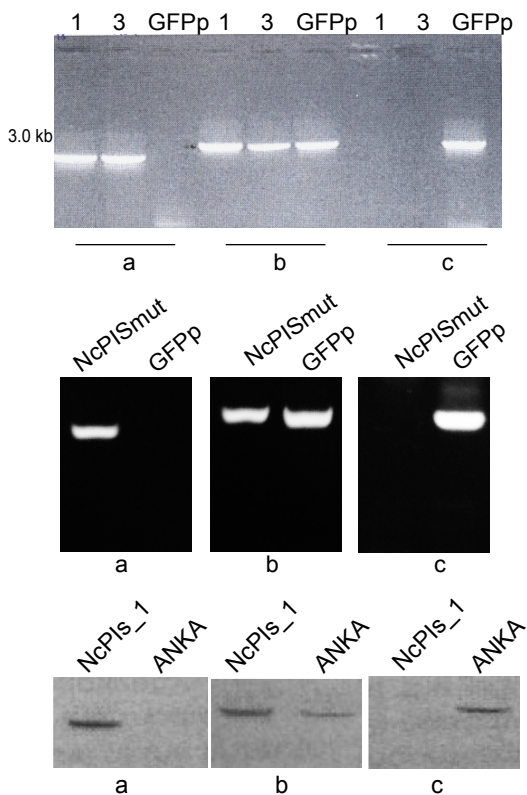

Supplement: S2 Fig — A. Schematic representation of the gene targeting strategy used for integrating NcPIS or NcPIsmut in the endogenous cssu-rrna locus. Double arrowhead line indicates the expected PCR product for an intact locus. Primers used for diagnostic PCR (L665, L260, L270 and L740) are indicated. B. PFIGE (Pulse-Field Inverted Gel Electrophoresis) analysis of transfected parasites. (i) DNA from transfected parasites run on 1% agarose gel, showing chromosomes separated according to their molecular size; Chromosomes 5/6 (which can’t be separated from each other) and 7 are indicated by arrows. (ii) The blot from the same gel hybridized with the 3’ UTR sequences of Pbdhfr/ts. The probe detects the 3’ UTR of the endogenous dhfr gene on chromosome 7, and the 3’ UTR of the selectable marker indicating correct integration on chromosome 5. C. PCR genotypic analysis of the derived transgenic clones. Upper panel: Lane 3 corresponds to NcPIS_3 clone. The primer pair a (L665/L740) amplifies the integration diagnostic 2.1kb band from genomic DNA isolated from all clones. Failure to amplify a 3kb band using the primer pair c (L270/L740) from gDNA of the two clones indicates that integration took place at the cssu-rrna locus, while presence of a 3kb band amplified by the primer set b (L260/L740) in WT control and the two NcPI-S expressing clones indicates the integrity of dssu-rrna locus. (Lower panel) The same primer sets were used to confirm integration of NcPISmut and NcPIS_1 in the specified locus. (PDF) [file pone.0121379.s002.pdf]

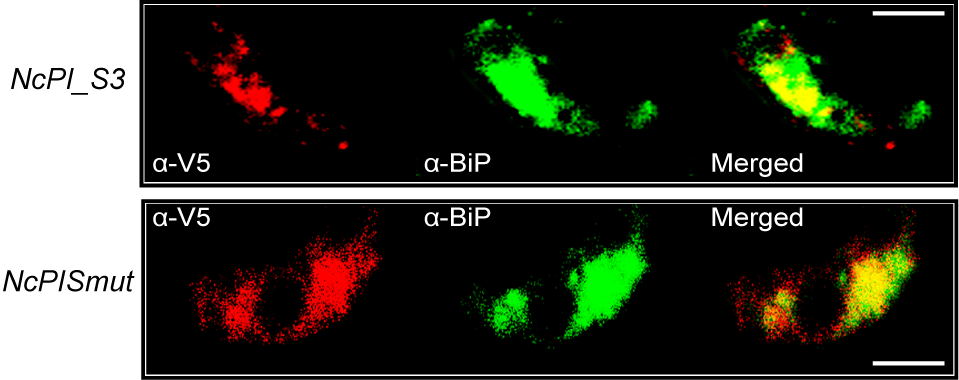

Supplement: S3 Fig — IFA analysis of NcPI_S3 and NcPI_Smut lines, showing expression of the PPI (red) and the ER-chaperone BiP (green) in P. berghei ookinetes. NcPI-Smut is expressed throughout the cytoplasm and the ER, while NcPI-S has limited colocalisation with BiP. Scale bar: 5 μM. (TIF) [file pone.0121379.s003.tif]

A

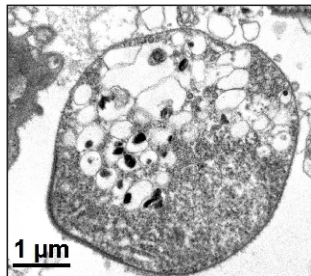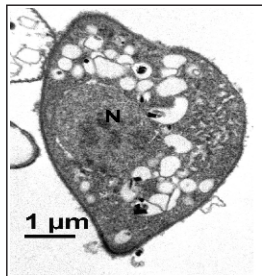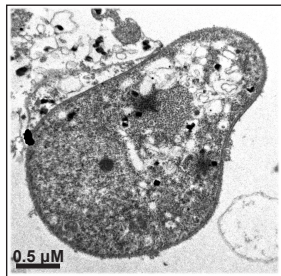

B

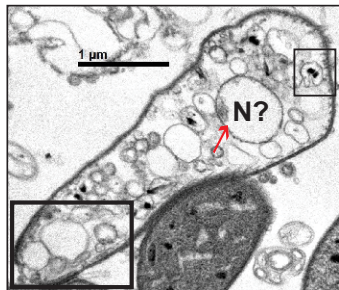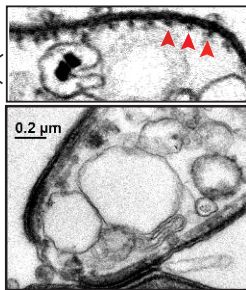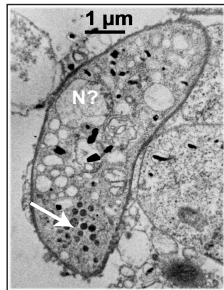

Supplement: S4 Fig — A. Developmentally arrested zygotes exhibiting the characteristic dilation of the endomembrane system alongside extensive vacuolation. B. Longitudinal sections of a sgIV ookinetes, in which the cytoplasm has been entirely replaced by electron-lucent vacuoles. Some subcellular structures, however, are still identifiable, such as subpellicular microtubules (arrowheads) and a well-structured collar with the characteristic electron-dense depositions surrounding the apical split; notice vacuole excretion from the apical split. A nucleus remnant (N) with a persistent electron dense area is defined by a rough envelope (red arrow). Reduced number of micronemes is also shown in one ookinete (white arrow). (PDF) [file pone.0121379.s004.pdf]

*ANKA*

*NcPIS\_1*

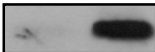

$\alpha$ -V5

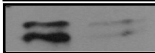

SOAP

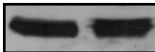

P28

Supplement: S5 Fig — Western analysis of extracts derived from in vitro ookinete cultures of NcPIS_1 and the parental ANKA 15cy1A (ANKA) strain, with V5 Mab (revealing NcPI-S) and antibodies against the micronemal protein SOAP, and the late zygote/ookinete specific P28 protein (sample normalization control). While the levels of P28 are similar between the parental ANKA strain and the NcPI-S clone, a reduction in the levels of SOAP is observed in the case of NcPIS_1 derived extracts. (PDF) [file pone.0121379.s005.pdf]
