# Supplementary material for: Ectopic Expression of a Neospora caninum Kazal Type Inhibitor Triggers Developmental Defects in Toxoplasma and Plasmodium
Source: PLoS One. 2015 Mar 24;10(3):e0121379. doi: 10.1371/journal.pone.0121379 (PMC4372514; doi:10.1371/journal.pone.0121379)
Supplement: S1 Table — (DOCX) [file pone.0121379.s006.docx]

**Primers used for DNA construct generation and genotype analysis (Sup. table 1).**

| **Primer Name**  NcPIS_5’  NcPIS_3’ ssRop1_5’  ssRop1_3’  myc(RI)_5’  myctoxo_3’  Ncmut_5’  Ncmut_3’  SPsub-2F  SPsub-2R  V5for  V5Rev  NcPIS_5’  NcPIS-R  NcPIsFor  NcPISRev  L665  L740  L270  L260 | **Oligonucleotide sequence** (introduced cloning sites are underlined)  GGCCGGCCCAAGGAAAATGAAG  TTAATTAATGTTTTTGCTTG  GATGCATGAGCAAAGGCTGC GGAATTCGGCGCTTGGGGTTG GGAATTCGAACAAAAACTCATCTC CTGGGCCGGCCCAGATCCTCTTC  aaggaaggccggcccaaggaaaatgaag  Ttaattaatgtttttgcttggatttatcc  GGATCCATGTTGAGAACA  AAGCTTAGATCTCTGGCCATTGTG  AGATCTGGTAAGCCTATCCCTAAC  AAGCTTGGCCGGCCCGTAGAATCG  GGCCGGCCCAAGGAAAATGAAG  GCGGCCGCTTAATTAATGTTTTTG  GGGCCGGCCCAAGGAAAATGAAGATCAGGGATGTATTTGCTTCGCGTCGGGCAAGCGTAGCTGCGGAACGGATGG  TTGCGGCCGCTTAATTAATGTTTTTGCTTGG  GTTGAAAAATTAAAAAAAAAC  CTAAGGTACGCATATCATGG  GTGTAGTAACATCAGTTATTGTGTG  ATACTGTATAACAGGTAAGCTGTTATTGTG |
| --- | --- |
